# Synthesis of novel benzenesulfamide derivatives with inhibitory activity against human cytosolic carbonic anhydrase I and II and *Vibrio cholerae* α- and β-class enzymes

**DOI:** 10.1080/14756366.2018.1467901

**Published:** 2018-07-10

**Authors:** Silvia Bua, Emanuela Berrino, Sonia Del Prete, Vallabhaneni S. Murthy, Vijayaparthasarathi Vijayakumar, Yasinalli Tamboli, Clemente Capasso, Elisabetta Cerbai, Alessandro Mugelli, Fabrizio Carta, Claudiu T. Supuran

**Affiliations:** a NEUROFARBA Department, Sezione di Scienze Farmaceutiche e Nutraceutiche, Università degli Studi di Firenze, Sesto Fiorentino (Florence), Italy;; b School of Advanced Sciences, Center for Organic and Medicinal Chemistry, VIT University, Vellore, India;; c Istituto di Bioscienze e Biorisorse, CNR, Napoli, Italy;; d Department of Neurosciences, Psychology, Drug's Research and Child's Health (NEUROFARBA), University of Florence, Firenze, Italy;; e Section of Pharmacology and Toxicology, Firenze, Italy

**Keywords:** Carbonic anhydrase inhibitors (CAIs), sulfamides, structure–activity relationship (SAR), *Vibrio cholerae*

## Abstract

The synthesis of a new series of sulfamides incorporating *ortho*-, *meta*, and *para*-benzenesulfamide moieties is reported, which were investigated for the inhibition of two human (h) isoforms of the zinc enzyme carbonic anhydrase (CA, EC 4.2.1.1), hCA I and II, and two *Vibrio cholerae* enzymes, belonging to the α- and β-CA classes (VchCAα, VchCAβ). The compounds were prepared by using the “tail approach”, aiming to overcome the scarcity of selective inhibition profiles associated to CA inhibitors belonging to the zinc binders. The built structure–activity relationship showed that the incorporation of benzhydryl piperazine tails on a phenyl sulfamide scaffold determines rather good efficacies against hCA I and VchCAα, with several compounds showing *K*_I_s < 100 nM. The activity was lower against hCA II and VchCAβ, probably due to the fact that the incorporated tails are quite bulky. The obtained evidences allow us to continue the investigations of different tails/zinc binding groups, with the purpose to increase the effectiveness/selectivity of such inhibitors against bacterial CAs from pathogens, affording thus potential new anti-infectives.

## 1. Introduction

Cholera is an infectious human disease of the small intestine and is caused by the gram-negative bacterium *Vibrio cholera*^1^^,^^2^. It is characterised by a massive loss of water and electrolytes which leads to severe dehydration and hypovolemic shock followed by death if the disease is not well treated^2–4^. In developing countries, cholera spreads among victims mainly through contaminated water sources, and countries without proper sanitation techniques have greater incidence of this disease^3^^,^^5^. In this regard, WHO reported 132,121 cases in 38 countries during 2016, which also included 2420 deaths^6^. It has been demonstrated that a potential inducer of virulence gene expression is sodium bicarbonate, which is present at a high concentration in the upper small intestine^2^. Consequently, bicarbonate is considered the first positive effector for ToxT, the major direct transcription activator of the virulence genes^2^.

Many studies conducted by some of us have shown that specific carbonic anhydrase (CA, EC 4.2.1.1) inhibitors, may control the bicarbonate-mediated virulence induction, suggesting the conversion of CO_2_ into bicarbonate by CA plays a crucial role as a virulence factor for *Vibrio cholerae*^7^^,^^8^. Three different CAs have been found in *Vibrio cholerae*, belonging to the three enzyme classes found in bacteria, VchCAα, β, and γ. VchCAs have been suggested as potential targets for anti-infectives development with a novel mechanism of action^9^^,^^10^.

Up to now, inhibition studies performed against VchCAs mainly considered sulfonamide-bearing derivatives, with sulfamates (R-OSO_2_NH_2_) and sulfamides (R-NHSO_2_NH_2_) being less investigated. These latter derivatives are the closest bioisosters and congeners to the primary sulfonamides (R-SO_2_NH_2_), which constitute the most important, clinically used class of CA inhibitors (CAIs)^11–14^.

We recently reported a set of benzhydrylpiperazine benzenesulfamide showing effective and selective inhibition profiles against the cytosolic isoform hCA I^15^. In the present study, we have oriented our efforts in developing sulfamide CAIs as potential antibacterial agents, continuing the development of the previously reported series of sulfamides^15^, and report the synthesis of new series of such derivatives. In addition to the bacterial enzyme VchCAα and β, these novel compounds were investigated for their property to inhibit the physiologically most important human cytosolic isoforms CA I and II^16^^,^^17^.

## 2. Materials and methods

### 2.1. General

Anhydrous solvents and all reagents were purchased from Sigma-Aldrich (Milan, Italy), Alfa Aesar (Milan, Italy), and TCI (Milan, Italy). All reactions involving air- or moisture-sensitive compounds were performed under a nitrogen atmosphere using dried glassware and syringes techniques to transfer solutions. Nuclear magnetic resonance spectra (^1^H NMR: 400 MHz; 13C NMR: 100 MHz; 19F NMR: 376 MHz) were recorded in DMSO-d_6_ using an Avance III 400 MHz spectrometer (Bruker, Milan, Italy). Chemical shifts are reported in parts per million (ppm) and the coupling constants (*J*) are expressed in Hertz (Hz). Splitting patterns are designated as follows: s, singlet; d, doublet; t, triplet; q, quadruplet; m, multiplet; brs, broad singlet; dd, double of doublets. The assignment of exchangeable protons (OH and NH) was confirmed by the addition of D_2_O. Analytical thin-layer chromatography (TLC) was carried out on silica gel F-254 plates (Merck, Milan, Italy). Melting points (m.p.) were carried out in open capillary tubes and are uncorrected. The solvents used in MS measures were acetone, acetonitrile (Chromasolv grade), purchased from Sigma-Aldrich (Milan, Italy) and mQ water 18 MX, obtained from Millipore’s Simplicity system (Milan, Italy). The mass spectra were obtained using a 1200 L triple quadrupole system (Varian, Palo Alto, CA) equipped by electrospray source (ESI) operating in both positive and negative ions. Stock solutions of analytes were prepared in acetone at 1.0 mg ml^−1^ and stored at 4 °C. Working solutions of each analyte were freshly prepared by diluting stock solutions in a mixture of mQ H_2_O/ACN 1:1 (v/v) up to a concentration of 1.0 µg ml^−1^. The mass spectra of each analyte were acquired by introducing, via syringe pump at 10 µl min^−1^, its working solution. Raw-data were collected and processed by Varian Workstation Vers. 6.8 software (Palo Alto, CA).

### 2.2. General procedure for the synthesis of compounds 2a–d

Compounds **1a**,**b** (1.0 eq) and the appropriate *N*-Boc-protected carboxylic acid (1.1 eq) in DMF (10.0 ml) were treated with DIPEA (2.0 eq), and HATU (1.5 eq) at r.t. for 30 min. When the reaction was complete (TLC: monitoring), it was quenched with ice cold water and extracted with ethyl acetate (3 × 15 ml). The combined organic layers were washed with H_2_O (3 × 15 ml), dried over Na_2_SO_4_, filtered-off and concentrated under reduced pressure to afford the title compounds **2a**–**d** as off-white solids.

#### 2.2.1. Tert-butyl (2-4-benzhydrylpiperazin-1-yl)-2-oxoethyl)carbamate (2a)

Using **1a** and *N*-Boc-glycine as starting materials, compound **2a** was obtained with yield: 99%, off white solid. ^1^H NMR (DMSO-d_6_, 400 MHz): *δ* 1.36 (9H, s, 3 × C*H*_3_), 2.27 (4H, m, 2 × piperazine-C*H*_2_), 3.42 (4H, m, 2 *×* piperazine-C*H*_2_), 3.72 (2H, d, *J* = 5.7, COC*H*_2_), 4.32 (1H, s, C*H*), 6.71 (1H, m, N*H*), 7.19 (2H, t, *J* = 7.2, 2 × Ar-*H*), 7.30 (4H, t, *J* = 7.5, 4 × Ar-*H*), 7.43 (4H, d, *J* = 7.4, Ar-*H*).

#### 2.2.2. Tert-butyl 4-4-benzhydrylpiperazine-1-carbonyl)piperidine-1-carboxylate (2b)

Using **1a** and *N*-Boc-isonipecotic acid as starting materials, compound **2b** was obtained with yield: 76%, off white solid. ^1^H NMR (DMSO-d_6_, 400 MHz): *δ* 1.38 (11H, s, 3 × C*H*_3_, piperidine-C*H*_2_), 1.55 (2H, m, piperidine-C*H*_2_), 2.25 (4H, br s, 2 × piperazine-C*H*_2_), 2.70 (3H, m, piperidine-C*H*_2_, COC*H*), 3.48 (4H, br d, 2 × piperazine-C*H*_2_), 3.87 (2H, m, piperidine-C*H*_2_), 4.40 (1H, s, C*H*), 7.19 (2H, t, *J* = 7.2, 2 × Ar-*H*), 7.30 (4H, t, *J* = 7.5, 4 × Ar-*H*), 7.43 (4H, d, *J* = 7.4, 4 × Ar-*H*).

#### 2.2.3. Tert-butyl (2-(4-(bis(4-fluorophenyl)methyl)piperazin-1-yl)-2-oxoethyl)carbamate (2c)

Using **1b** and *N*-Boc-glycine as starting materials, compound **2c** was obtained with yield: 99%, off white solid. ^1^H NMR (DMSO-d_6_, 400 MHz): *δ* 1.36 (9H, s, 3 × C*H*_3_), 2.27 (4H, m, 2 × piperazine-C*H*_2_), 3.42 (4H, m, 2 *×* piperazine-C*H*_2_), 3.72 (2H, d, *J* = 5.7, COC*H*_2_), 4.32 (1H, s, C*H*), 6.71 (1H, m, N*H*), 7.14 (4H, m, *J* = 7.2, 4 × Ar-*H*), 7.43 (4H, m, *J* = 7.4, 4 × Ar-*H*).

#### 2.2.4. Tert-butyl 4-(4-(bis(4-fluorophenyl)methyl)piperazine-1-carbonyl)piperidine-1-carboxylate (2d)

Using **1b** and *N*-Boc-isonipecotic acid as starting materials, compound **2d** was obtained with yield: 71%, off white solid. ^1^H NMR (DMSO-d_6_, 400 MHz): *δ* 1.38 (11H, s, 3 × C*H*_3_, piperidine-C*H*_2_), 1.55 (2H, m, piperidine-C*H*_2_), 2.25 (4H, br s, 2 × piperazine-C*H*_2_), 2.70 (3H, m, piperidine-C*H*_2_, COC*H*), 3.48 (4H, br d, 2 × piperazine-C*H*_2_), 3.87 (2H, m, piperidine-C*H*_2_), 4.40 (1H, s, C*H*), 7.14 (4H, t, *J* = 8.7, 4 × Ar-*H*), 7.44 (4H, t, *J* = 8.1, 4 × Ar-*H*).

#### 2.2.5. General procedure for the synthesis of compounds 4a–p

A stirred solution of compounds **2a**–**d** (1.0 eq) in DCM (10.0 ml) was treated with TFA (3.0 eq) and stirred at r.t. for 2 h. The reaction mixture was concentrated to dry and co-distilled twice with DCM to afford the corresponding amines **3a–d** as TFA salts (not isolated), which were readily dissolved in DCM (10.0 ml) and treated with DIPEA (5.0 eq) and the appropriate sulfonyl chloride (1.2 eq). The reaction solutions were stirred at r.t. for 1 h, then concentrated to dry and the residue obtained was purified by silica gel column chromatography using ethyl acetate in *n*-hexane (20–40% v/v) as eluent to afford the titled compounds **4a**–**p** as off-white solids.

#### 2.2.6. 1-Benzhydryl-4-((3-nitrophenyl)sulfonyl)piperazine (4a)

Using **1a** and 3-nitrobenzenesulfonyl chloride as starting materials, compound **4a** was obtained with yield 75%, off white solid; m.p. 170–172 °C; ^1^H NMR (DMSO-d_6_, 400 MHz): *δ* 2.36 (4H, br s, 2 × piperazine-C*H*_2_), 3.00 (4H, br s, 2 × piperazine-C*H*_2_), 4.31 (1H, s, C*H*), 7.14 (2H, t, *J* = 7.2, 2 × Ar-*H*), 7.23 (4H, t, *J* = 7.4, 4 × Ar-*H*), 7.33 (4H, d, *J* = 7.3, 4 × Ar-*H*), 7.96 (1H, t, *J* = 8.0, Ar-*H*), 8.15 (1H, d, *J* = 7.8, Ar-*H*), 8.35 (1H, s, Ar-*H*), 8.56 (1H, m, Ar-*H*); 13C NMR (DMSO-d_6_, 100 MHz): *δ* 46.0, 50.3, 74.1, 122.2, 126.9, 127.4, 127.8, 128.5, 131.5, 133.4, 136.5, 142.2, 148.2; MS (ESI positive) *m/z* = 437.9 [M + H]^+^.

#### 2.2.7. 1-Benzhydryl-4-((4-nitrophenyl)sulfonyl)piperazine (4b)

Using **1a** and 4-nitrobenzenesulfonyl chloride as starting materials, compound **4b** was obtained with yield 46%, off white solid; m.p. 216–218 °C; ^1^H NMR (DMSO-d_6_, 400 MHz): *δ* 2.36 (4H, br s, 2 × piperazine-C*H*_2_), 2.99 (4H, br s, 2 × piperazine-C*H*_2_), 4.31 (1H, s, C*H*), 7.14 (2H, t, *J* = 7.2, 2 × Ar-*H*), 7.23 (4H, t, *J* = 7.4, 4 × Ar-*H*), 7.33 (4H, d, *J* = 7.3, 4 × Ar-*H*), 7.98 (2H, d, *J* = 8.8, 2 × Ar-*H*), 8.45 (2H, d, *J* = 8.8, 2 × Ar-*H*); ^13^C NMR (DMSO-d_6_, 100 MHz): *δ* 46.0, 50.2, 74.0, 124.7, 126.9, 127.4, 128.5, 129.1, 140.5, 142.1, 150.1; MS (ESI positive) *m/z* = 437.9 [M + H]^+^.

#### 2.2.8. N-(2-(4-Benzhydrylpiperazin-1-yl)-2-oxoethyl)-2-nitrobenzenesulfonamide (4c)

Using **3a** and 2-nitrobenzenesulfonyl chloride as starting materials, compound **4c** was obtained with yield 61%, off white solid; m.p. 188–192 °C; ^1^H NMR (DMSO-d_6_, 400 MHz): *δ* 2.20 (4H, br d, 2 × piperazine-C*H*_2_), 3.37 (4H, br s, 2 × piperazine-C*H*_2_), 3.89 (2H, d, *J* = 4.8, *CH_2_*), 4.28 (1H, s, C*H*), 7.17 (2H, t, *J* = 7.3, 2 × Ar-*H*), 7.28 (4H, t, *J* = 7.4, 4 × Ar-*H*), 7.40 (4H, d, *J* = 7.3, 4 × Ar-*H*), 7.81 (2H, m, 2 Ar-*H*), 7.93 (2H, m, Ar-*H*, N*H*), 8.01 (1H, m, Ar-*H*); ^13^C NMR (DMSO-d_6_, 100 MHz): *δ* 41.5, 43.9, 44.0, 50.9, 51.3, 74.6, 124.4, 126.9, 127.5, 128.5, 129.8, 132.6, 133.1, 133.8, 142.3, 147.4, 165.4; MS (ESI positive) *m/z* = 494.9 [M + H]^+^.

#### 2.2.9. N-(2-(4-Benzhydrylpiperazin-1-yl)-2-oxoethyl)-3-nitrobenzenesulfonamide (4d)

Using **3a** and 3-nitrobenzenesulfonyl chloride as starting materials, compound **4d** was obtained with yield 56%, off white solid; m.p. 208–212 °C; ^1^H NMR (DMSO-d_6_, 400 MHz): *δ* 2.13 (2H, s, piperazine-C*H*_2_), 2.20 (2H, s, piperazine-C*H*_2_), 3.30 (4H, br s, 2 × piperazine-C*H*_2_), 3.81 (2H, d, *J* = 5.5, C*H*_2_), 4.24 (1H, s, C*H*), 7.17 (2H, t, *J* = 7.3, 2 × Ar-*H*), 7.28 (4H, t, *J* = 7.4, 4 × Ar-*H*), 7.39 (4H, d, *J* = 7.2, 4 × Ar-*H*), 7.84 (1H, t, *J* = 8.0, Ar-*H*), 8.17 (2H, m, Ar-*H*, N*H*), 8.43 (1H, m, Ar-*H*), 8.54 (1H, m, Ar-*H*); ^13^C NMR (DMSO-d_6_, 100 MHz): *δ* 41.4, 43.6, 44.1, 50.9, 51.5, 74.7, 121.5, 126.8, 126.9, 127.5, 128.5, 130.9, 132.8, 142.3, 147.6, 165.3; MS (ESI positive) *m/z* = 494.9 [M + H]^+^.

#### 2.2.10. (4-Benzhydrylpiperazin-1-yl)(1-((2-nitrophenyl)sulfonyl)pipe-ridin-4-yl)methanone (4e)

Using **3b** and 2-nitrobenzenesulfonyl chloride as starting materials, compound **4e** was obtained with yield 49%, off white solid; m.p. 158–160 °C; ^1^H NMR (DMSO-d_6_, 400 MHz): *δ* 1.48 (2H, m, piperidine-C*H*_2_), 1.66 (2H, m, piperidine-C*H*_2_), 2.22 (4H, br s, 2 × piperazine-C*H*_2_), 2.72 (3H, m, piperidine-C*H*_2_, COC*H*), 3.44 (4H, br d, 2 × piperazine-C*H*_2_), 3.66 (2H, d, piperidine-C*H*_2_), 4.29 (1H, s, C*H*), 7.17 (2H, t, *J* = 7.3, 2 × Ar-*H*), 7.28 (4H, t, *J* = 7.4, 4 × Ar-*H*), 7.40 (4H, d, *J* = 7.5, 4 × Ar-*H*), 7.85 (2H, m, 2 × Ar-*H*), 7.97 (2H, m, 2 × Ar-*H*); ^13^C NMR (DMSO-d_6_, 100 MHz): *δ* 27.7, 35.7, 41.3, 44.7, 45.0, 51.2, 52.0, 074.6, 124.0, 126.9, 127.5, 128.5, 129.2, 130.3, 132.1, 134.7, 142.4, 147.8, 171.6; MS (ESI positive) *m/z* = 548.9 [M + H]^+^.

#### 2.2.11. (4-Benzhydrylpiperazin-1-yl)(1-((3-nitrophenyl)sulfonyl)pipe-ridin-4-yl)methanone (4f)

Using **3b** and 3-nitrobenzenesulfonyl chloride as starting materials, compound **4f** was obtained with yield 48%, off white solid; m.p. 222–226 °C; ^1^H NMR (DMSO-d_6_, 400 MHz): *δ* 1.52 (2H, m, piperidine-C*H*_2_), 1.64 (2H, m, piperidine-C*H*_2_), 2.20 (4H, br s, 2 × piperazine-C*H*_2_), 2.37 (2H, m, piperidine-C*H*_2_), 2.53 (1H, m, COC*H*), 3.41 (4H, 4 s, 2 × piperazine-C*H*_2_), 3.63 (2H, d, piperidine-C*H*_2_), 4.27 (1H, s, C*H*), 7.17 (2H, t, *J* = 7.3, 2 × Ar-*H*), 7.27 (4H, t, *J* = 7.4, 4 × Ar-*H*), 7.39 (4H, d, *J* = 7.5, 4 × Ar-*H*), 7.93 (1H, t, *J* = 8.0, Ar-*H*), 8.16 (1H, d, *J* = 7.8, Ar-*H*), 8.34 (1H, br s, Ar-*H*), 8.52 (1H, d, *J* = 8.1, Ar-*H*); ^13^C NMR (DMSO-d_6_, 100 MHz): *δ* 27.5, 35.6, 41.1, 44.6, 45.2, 51.2, 52.0, 74.6, 121.8, 126.8, 126.9, 127.6, 128.5, 131.5, 133.4, 137.1, 142.3, 147.9, 171.5; MS (ESI positive) *m/z* = 548.9 [M + H]^+^.

#### 2.2.12. (4-Benzhydrylpiperazin-1-yl)(1-((4-nitrophenyl)sulfonyl)pipe-ridin-4-yl)methanone (4g)

Using **3b** and 4-nitrobenzenesulfonyl chloride as starting materials, compound **4g** was obtained with yield 59%, off white solid; m.p. 182–186 °C; ^1^H NMR (DMSO-d_6_, 400 MHz): *δ* 1.49 (2H, m, piperidine-C*H*_2_), 1.64 (2H, m, piperidine-C*H*_2_), 2.20 (4H, br s, 2 × piperazine-C*H*_2_), 2.37 (2H, m, piperidine-C*H*_2_), 2.57 (1H, m, COC*H*), 3.41 (4H, br s, 2 × piperazine-C*H*_2_), 3.62 (2H, m, piperidine-C*H*_2_), 4.27 (1H, s, C*H*), 7.16 (2H, t, *J* = 7.3, 2 × Ar-*H*), 7.27 (4H, t, *J* = 7.4, 4 × Ar-*H*), 7.39 (4H, d, *J* = 7.5, 4 × Ar-*H*), 7.99 (2H, d, *J* = 6.9, 2 × Ar-*H*), 8.42 (2H, d, *J* = 6.9, 2 × Ar-*H*); ^13^C NMR (DMSO-d_6_, 100 MHz): *δ* 26.5, 35.6, 41.1, 44.7, 45.1, 51.2, 51.9, 74.6, 124.6, 126.9, 127.5, 128.5, 128.9, 141.2, 142.3, 149.9, 171.5; MS (ESI positive) *m/z* = 548.9 [M + H]^+^.

#### 2.2.13. 1-(Bis(4-Fluorophenyl)methyl)-4-((2-nitrophenyl)sulfonyl)pip-erazine (4h)

Using **1b** and 2-nitrobenzenesulfonyl chloride as starting materials, compound **4h** was obtained with yield: 77%, off white solid; m.p. 120–124 °C; ^1^H NMR (DMSO-d_6_, 400 MHz): *δ* 2.34 (4H, br s, 2 × piperazine-C*H*_2_), 3.18 (4H, br s, 2 × piperazine-C*H*_2_), 4.43 (1H, s, C*H*), 7.09 (4H, t, *J* = 8.7, 4 × Ar-*H*), 7.39 (4H, t, *J* = 8.2, 4 Ar-*H*), 7.91 (4H, m, 4 × Ar-*H*); ^13^C NMR (DMSO-d_6_, 100 MHz): *δ* 45.9, 50.3, 72.0, 115.2, 115.4, 124.1, 128.4, 129.3, 129.4, 130.4, 132.2, 134.9, 138.1, 147.9, 159.9, 162.3; MS (ESI positive) *m/z* = 474.04 [M + H]^+^.

#### 2.2.14. 1-(Bis(4-Fluorophenyl)methyl)-4-((3-nitrophenyl)sulfonyl)pip-erazine (4i)

Using **1b** and 3-nitrobenzenesulfonyl chloride as starting materials, compound **4i** was obtained with yield: 87%, off white solid; m.p. 168–172 °C; ^1^H NMR (DMSO-d_6_, 400 MHz): *δ* 2.34 (4H, br s, 2 × piperazine-C*H*_2_), 2.99 (4H, br s, 2 × piperazine-C*H*_2_), 4.40 (1H, s, C*H*), 7.07 (4H, t, *J* = 8.8, 4 × Ar-*H*), 7.35 (4H, q, *J* = 5.7, 4 × Ar-*H*), 7.96 (1H, t, *J* = 8.0, Ar-*H*), 8.15 (1H, d, *J* = 7.8, Ar-*H*), 8.35 (1H, s, Ar-*H*), 8.56 (1H, d, *J* = 8.1, Ar-*H*); ^13^C NMR (DMSO-d_6_, 100 MHz): *δ* 45.9, 50.0, 71.9, 115.2, 115.4, 124.7, 129.1, 129.2, 129.4, 138.1, 143.5, 155.1, 159.8, 162.2; MS (ESI positive) *m/z* = 473.9 [M + H]^+^.

#### 2.2.15. 1-(Bis(4-Fluorophenyl)methyl)-4-((4-nitrophenyl)sulfonyl)pip-erazine (4j)

Using **1b** and 4-nitrobenzenesulfonyl chloride as starting materials, compound **4j** was obtained with yield: 73%, yellow solid; m.p. 222–226 °C; ^1^H NMR (DMSO-d_6_, 400 MHZ): *δ* 2.34 (4H, br s, 2 × piperazine-C*H*_2_), 2.99 (4H, br s, 2 × piperazine-C*H*_2_), 4.40 (1H, s, C*H*), 7.07 (4H, t, *J* = 8.8, 4 Ar-*H*), 7.34 (4H, m, *J* = 5.7, 4 × Ar-*H*), 7.98 (2H, d, *J* = 8.7, 2 × Ar-*H*), 8.45 (2H, d, *J* = 8.7, 2 × Ar-*H*); ^13^C NMR (DMSO-d_6_, 100 MHz): *δ* 46.0, 50.0, 71.9, 115.2, 115.4, 124.7, 129.1, 129.2, 129.3, 138.1, 140.5, 150.1, 159.8, 162.2; MS (ESI positive) *m/z* = 473.9 [M + H]^+^.

#### 2.2.16. N-(2-(4-(bis(4-Fluorophenyl)methyl)piperazin-1-yl)-2-oxoethyl)-2-nitrobenzenesulfonamide (4k)

Using **3c** and 2-nitrobenzenesulfonyl chloride as starting materials, compound **4k** was obtained with yield: 69%, off white solid; m.p. 136–140 °C; ^1^H NMR (DMSO-d_6_, 400 MHz): *δ* 2.20 (4H, br d, 2 × pipeazine-C*H*_2_), 3.37 (4H, br s, 2 × piperazine-C*H*_2_), 3.91 (2H, d, *J* = 5.38, C*H*_2_), 4.39 (1H, s, C*H*), 7.13 (4H, t, *J* = 8.7, 4 × Ar-*H*), 7.42 (4H, q, *J* = 5.8, 4 × Ar-*H*), 7.83 (2H, m, 2 × Ar-*H*), 8.02 (3H, m, Ar-H, N*H*), 8.01 (1H, m, Ar-*H*); ^13^C NMR (DMSO-d_6_, 100 MHz): *δ* 41.5, 43.9, 43.9, 50.9, 51.3, 72.5, 115.2, 115.5, 124.4, 129.4, 129.5, 129.9, 132.6, 133.1, 133.9, 138.3, 159.5, 162.7, 165.4; MS (ESI positive) *m/z* = 531.12 [M + H]^+^.

#### 2.2.17. N-(2-(4-(bis(4-Fluorophenyl)methyl)piperazin-1-yl)-2-oxoethyl)-3-nitrobenzenesulfonamide (4l)

Using **3c** and 3-nitrobenzenesulfonyl chloride as starting materials, compound **4l** was obtained with yield: 58%, off white solid; m.p. 202–206 °C; ^1^H NMR (DMSO-d_6_, 400 MHz): *δ* 2.15 (4H, br s, 2 × piperazine-C*H*_2_), 3.30 (4H, br s, 2 × piperazine-C*H*_2_), 3.82 (2H, d, *J* = 5.16, C*H*_2_), 4.34 (1H, s, C*H*), 7.11 (4H, t, *J* = 8.7, 4 × Ar-*H*), 7.40 (4H, t, *J* = 6.1, 4 × Ar-*H*), 7.84 (1H, t, *J* = 8.01, Ar-*H*), 8.15 (1H, t, *J* = 5.09, N*H*), 8.19 (1H, d, *J* = 7.78, Ar-*H*), 8.44 (1H, d, *J* = 8.16, Ar-*H*), 8.53 (1H, s, Ar-*H*); ^13^C NMR (DMSO-d_6_, 100 MHz): *δ* 23.6, 26.6, 37.3, 41.1, 44.8, 45.9, 48.1, 51.0, 51.8, 72.6, 115.3, 115.6, 124.5, 129.4, 129.5, 129.9, 132.6, 133.2, 133.8, 138.3, 159.5, 162.8, 165.5; MS (ESI positive) *m/z* = 531.04 [M + H]^+^.

#### 2.2.18. N-(2-(4-(bis(4-Fluorophenyl)methyl)piperazin-1-yl)-2-oxoethyl)-4-nitrobenzenesulfonamide (4m)

Using **3c** and 4-nitrobenzenesulfonyl chloride as starting materials, compound **4m** was obtained with yield: 47%, off white solid; m.p. 176–180 °C; ^1^H NMR (DMSO-d_6_, 400 MHz): *δ* 2.17 (4H, br d, 2 × piperazine-C*H*_2_), 3.33 (4H, br s, 2 × piperazine-C*H*_2_), 3.81 (2H, d, *J* = 5.5, C*H*_2_), 4.37 (1H, s, C*H*), 7.11 (4H, t, *J* = 8.6, 4 × Ar-*H*), 7.41 (4H, t, *J* = 6.1, 4 × Ar-*H*), 8.03 (2H, d, *J* = 8.6, 2 × Ar-*H*), 8.15 (1H, t, *J* = 5.4, N*H*), 8.36 (2H, d, *J* = 8.6, 2 × Ar-*H*); ^13^C NMR (DMSO-d_6_, 100 MHz): *δ* 41.4, 43.7, 44.1, 50.7, 51.2, 72.5, 115.5, 124.2, 129.3, 132.4, 138.2, 146.3, 149.4, 159.8, 162.3, 165.3; MS (ESI positive) *m/z* = 531.04 [M + H]^+^.

#### 2.2.19. (4-(bis(4-Fluorophenyl)methyl)piperazin-1-yl)(1-((2-nitrophenyl)sulfonyl)piperidin-4-yl)methanone (4n)

Using **3d** and 2-nitrobenzenesulfonyl chloride as starting materials, compound **4n** was obtained with yield: 59%, off white solid; m.p. 170–174 °C; ^1^H NMR (DMSO-d_6_, 400 MHz): *δ* 1.48 (2H, m, piperidine-C*H*_2_), 1.66 (2H, m, piperidine-C*H*_2_), 2.20 (4H, br s, 2 × piperazine-C*H*_2_), 2.71 (3H, m, piperidine-C*H*_2_, COC*H*), 3.44 (4H, br d, 2 × piperazine-C*H*_2_), 3.67 (2H, m, piperidine-C*H*_2_), 4.38 (1H, s, C*H*), 7.11 (4H, t, *J* = 8.7, 4 × Ar-*H*), 7.41 (4H, m, 4 × Ar-*H*), 7.91 (4H, m, 4 × Ar-*H*); ^13^C NMR (DMSO-d_6_, 100 MHz): *δ* 27.7, 35.7, 40.1, 44.8, 44.9, 51.0, 51.8, 72.5, 115.2, 115.4, 124.0, 129.2, 129.3, 129.4, 130.3, 132.1, 134.7, 138.3, 147.8, 159.8, 162.2, 171.6; MS (ESI positive) *m/z* = 585.11 [M + H]^+^.

#### 2.2.20. (4-(bis(4-Fluorophenyl)methyl)piperazin-1-yl)(1-((3-nitrophenyl)sulfonyl)piperidin-4-yl)methanone (4o)

Using **3d** and 3-nitrobenzenesulfonyl chloride as starting materials, compound **4o** was obtained with yield: 48%, off white solid; m.p. 180–184 °C; ^1^H NMR (DMSO-d_6_, 400 MHz): *δ* 1.52 (2H, m, piperdine-C*H*_2_), 1.65 (2H, d, piperidine-C*H*_2_), 2.20 (4H, s, 2 × piperazine-C*H*_2_), 2.37 (2H, m, piperidne-C*H*_2_), 2.54 (1H, m, COC*H*), 3.41 (4H, br s, 2 × piperazine-C*H*_2_), 3.64 (2H, m, piperidine-C*H*_2_), 4.29 (1H, s, C*H*), 7.12 (4H, t, *J* = 8.7, 4 × Ar-*H*), 7.43 (4H, t, *J* = 5.7, 4 × Ar-*H*), 7.94 (1H, t, *J* = 8.7, Ar-*H*), 8.16 (1H, d, *J* = 8.8, Ar-*H*), 8.34 (1H, s, Ar-*H*), 8.53 (1H, d, *J* = 8.7, Ar-*H*); ^13^C NMR (DMSO-d_6_, 100 MHz): *δ* 27.5, 35.7, 41.1, 44.7, 45.2, 51.2, 51.9, 74.6, 115.3, 115.5, 121.9, 127.8, 129.4, 129.3, 129.4, 129.5, 131.5, 133.3, 137.5, 138.4, 138.4, 148.1, 159.5, 162.7, 170.6; MS (ESI positive) *m/z* = 585.09 [M + H]^+^.

#### 2.2.21. (4-(bis(4-Fluorophenyl)methyl)piperazin-1-yl)(1-((4-nitrophenyl)sulfonyl)piperidin-4-yl)methanone (4p)

Using **3d** and 4-nitrobenzenesulfonyl chloride as starting materials, compound **4p** was obtained with yield: 46%, off white solid; m.p. 232–236 °C; ^1^H NMR (DMSO-d_6_, 400 MHz): *δ* 1.54 (2H, m, piperidine-C*H*_2_), 1.66 (2H, m, C*H*_2_), 2.21 (4H, br s, 2 × piperazine-C*H*_2_), 2.37 (2H, m, piperidine-C*H*_2_), 2.80 (1H, m, COC*H*), 3.41 (4H, br s, 2 × piperazine-C*H*_2_), 3.65 (2H, m, piperdine-C*H*_2_), 4.30 (1H, s, C*H*), 7.12 (4H, t, *J* = 8.8, 4 × Ar-*H*), 7.44 (4H, t, *J* = 5.7, 4 × Ar-*H*), 7.98 (2H, d, *J* = 9.2, 2 × Ar-*H*) 8.42 (2H, d, *J* = 9.2, 2 × Ar-*H*); ^13^C NMR (DMSO-d_6_, 100 MHz): *δ* 26.6, 35.6, 41.2, 44.7, 45.2, 51.3, 51.9, 74.7, 115.2, 115.4, 124.7, 128.7, 129.3, 129.4, 138.3, 141.4, 149.9, 159.8, 162.3, 170.3; MS (ESI positive) *m/z* = 585.10 [M + H]^+^.

#### 2.2.22. General procedure for the synthesis of compounds 5a–p

The appropriate nitrobenzenesulfonamides **4a–p** (1.0 eq) in a solution of H_2_O (0.4 ml) and EtOH (0.3 ml) was treated with glacial AcOH (0.05 ml) and Fe (0) (12.0 eq). The reaction mixture was stirred at 75 °C for 1 h (TLC monitoring), then cooled to r.t. and diluted with EtOAc (10.0 ml). The mixture was filtered through Celite 521^®^, washed with a saturated NaHCO_3_ aqueous solution (3 × 15 ml), brine (3 × 10 ml) and dried over Na_2_SO_4_. The organic solvent was evaporated *in vacuo* to give an oil residue, which was triturated from Et_2_O, to afford the titled compounds **5a–p** as white solids.

#### 2.2.23. 3-((4-Benzhydrylpiperazin-1-yl)sulfonyl)aniline (5a)

Compound **5a** was obtained in 70% yield; m.p. 120–122 °C; TLC: *R_f_* = 0.17 (ethyl acetate/*n-*hexane 20% v/v); ^1^H NMR (DMSO-d_6_, 400 MHZ) (DMSO-d_6_, 400 MHZ) (DMSO-d_6_, 400 MHz): *δ* 2.40 (4H, m, 2 × piperazine-C*H*_2_), 2.92 (4H, m, 2 × piperazine-C*H*_2_), 4.34 (1H, s, C*H*), 5.70 (2H, s, exchange with D_2_O, N*H*_2_), 6.81 (1H, m, Ar-*H*), 6.91 (2H, m, Ar-*H*), 7.20 (2H, t, *J* = 7.2, Ar-*H*), 7.29 (5H, m, Ar-*H*), 7.40 (4H, m, Ar-*H*); ^13^C NMR (DMSO-d_6_, 100 MHz): *δ* 47.0, 51.4, 75.3, 114.5, 117.3, 118.4, 127.9, 128.4, 129.4, 130.9, 138.0, 142.7, 148.2; MS (ESI positive) *m/z* = 408.2 [M + H]^+^.

#### 2.2.24. 4-((4-Benzhydrylpiperazin-1-yl)sulfonyl)aniline (5b)

Compound **5b** was obtained in 80% yield; m.p. 196–198 °C; TLC: *R_f_* = 0.25 (ethyl acetate/*n-*hexane 20% v/v); ^1^H NMR (DMSO-d_6_, 400 MHz): *δ* 2.38 (4H, m, 2 × piperazine-C*H*_2_), 2.85 (4H, m, 2 × piperazine-C*H*_2_), 4.33 (1H, s, C*H*), 6.16 (2H, s, exchange with D_2_O, N*H*_2_), 6.70 (2H, m, Ar-*H*), 7.20 (2H, m, Ar-*H*), 7.29 (4H, t, *J* = 7.4, Ar-*H*), 7.40 (6H, m, Ar-*H*); ^13^C NMR (DMSO-d_6_, 100 MHz): *δ* 47.0, 51.4, 75.3, 113.6, 126.8, 127.8, 128.4, 129.3, 130.6, 143.4, 154.2; MS (ESI positive) *m/z* = 408.2 [M + H]^+^.

#### 2.2.25. 2-Amino-N-(2-(4-benzhydrylpiperazin-1-yl)-2-oxoethyl)benzenesulfonamide (5c)

Compound **5c** was obtained in 85% yield; m.p. 140–142 °C; TLC: *R_f_* = 0.39 (ethyl acetate/*n-*hexane 50% v/v); ^1^H NMR (DMSO-d_6_, 400 MHz): *δ* 2.25 (4H, m, 2 × piperazine-C*H*_2_), 3.42 (4H, m, 2 × piperazine-C*H*_2_), 3.66 (2H, d, *J* = 5.4, COC*H_2_*NH), 4.33 (1H, s, C*H*), 5.99 (2H, s, exchange with D_2_O, N*H*_2_), 6.59 (1H, t, *J* = 7.4, Ar-*H*), 6.80 (1H, d, *J* = 8.0, Ar-*H*), 7.24 (3H, m, Ar-*H*), 7.33 (4H, m, Ar-*H*), 7.47 (6H, m, Ar-*H*); ^13^C NMR (DMSO-d_6_, 100 MHz): *δ* 42.4, 45.1, 52.2, 75.5, 115.7, 117.8, 120.3, 127.8, 128.4, 129.4, 130.0, 134.4, 143.3, 147.3, 166.43; MS (ESI positive) *m/z* = 465.2 [M + H]^+^.

#### 2.2.26. 3-Amino-N-(2-(4-benzhydrylpiperazin-1-yl)-2-oxoethyl)benzenesulfonamide (5d)

Compound **5d** was obtained in 78% yield; m.p. 135–137 °C; TLC: *R_f_* = 0.33 (ethyl acetate/*n-*hexane 50% v/v); ^1^H NMR (DMSO-d_6_, 400 MHz): *δ* 2.26 (4H, m, 2 × piperazine-C*H*_2_), 3.33 (4H, overlap with water peak, 2 × piperazine-C*H*_2_), 3.67 (2H, s, COC*H_2_*NH), 4.34 (1H, s, C*H*), 5.58 (2H, s, exchange with D_2_O, N*H*_2_), 6.76 (1H, d, *J* = 6.8, Ar-*H*), 6.90 (1H, d, *J* = 7.0, Ar-*H*), 6.99 (1H, m, Ar-*H*), 7.21 (3H, m, Ar-*H*), 7.33 (4H, m, Ar-*H*), 7.45 (5H, m, 4 × Ar-*H*, exchange with D_2_O, CH_2_N*H*SO_2_); ^13^C NMR (DMSO-d_6_, 100 MHz): *δ* 42.2, 44.9, 51.9, 75.7, 116.4, 120.7, 122.5, 126.2, 127.6, 128.2, 129.4, 141.1, 141.6, 148.3, 164.3; MS (ESI positive) *m/z* = 465.2 [M + H]^+^.

#### 2.2.27. (1-((2-Aminophenyl)sulfonyl)piperidin-4-yl)(4-benzhydrylpiperazin-1-yl)methanone (5e)

Compound **5e** was obtained in 47% yield; m.p. 202–234 °C; TLC: *R_f_* = 0.54 (ethyl acetate/*n-*hexane 60% v/v); ^1^H NMR (DMSO-d_6_, 400 MHz): *δ* 1.52 (2H, m, piperidine-C*H*_2_), 1.67 (2H, m, piperidine-C*H*_2_), 2.26 (4H, m, 2 × piperazine-C*H*_2_), 2.54 (3H, overlap with DMSO peak, COC*H*, piperidine-C*H*_2_), 3.47 (4H, m, 2 × piperazine-C*H*_2_), 3.63 (2H, m, piperidine-C*H*_2_), 4.32 (1H, s, C*H*), 6.07 (2H, s, exchange with D_2_O, N*H*_2_), 6.65 (1H, m, Ar-*H*), 6.86 (1H, d, *J* = 7.6, Ar-*H*), 7.22 (2H, m, Ar-*H*), 7.32 (5H, m, Ar-*H*), 7.38 (1H, t, *J* = 7.6, Ar-*H*) 7.42 (4H, m, Ar-*H*); ^13^C NMR (DMSO-d_6_, 100 MHz): *δ* 28.6, 36.8, 42.0, 45.9, 53.0, 75.6, 116.6, 127.8, 128.5, 129.4, 130.9, 132.5, 134.6, 134.7, 135.5, 143.3, 167.8; MS (ESI positive) *m/z* = 519.7 [M + H]^+^.

#### 2.2.28. (1-((3-Aminophenyl)sulfonyl)piperidin-4-yl)(4-benzhydrylpiperazin-1-yl)methanone (5f)

Compound **5f** was obtained in 30% yield; m.p. 124–126 °C; TLC: *R_f_* = 0.13 (ethyl acetate/*n-*hexane 60% v/v); ^1^H NMR (DMSO-d_6_, 400 MHz): *δ* 1.54 (2H, m, piperidine-C*H*_2_), 1.68 (2H, m, piperidine-C*H*_2_), 2.29 (6H, m, 2 × piperazine-C*H*_2_, piperidine-C*H*_2_), 2.54 (1H, overlap with DMSO peak, COC*H*) 3.33 (4H, m, overlap with water peak, 2 × piperazine-C*H*_2_), 3.56 (2H, m, piperidine-C*H*_2_), 4.32 (1H, s, C*H*), 5.64 (2H, s, exchange with D_2_O, N*H*_2_) 6.81 (2H, m, Ar-*H*), 6.91 (1H, m, Ar-*H*), 7.23 (3H, m, Ar-*H*), 7.30 (4H, t, *J* = 7.6, Ar-*H*), 7.44 (4H, d, *J* = 7.2, Ar-*H*); ^13^C NMR (DMSO-d_6_, 100 MHz): *δ* 28.6, 36.8 (overlap with DMSO peak), 40.4 (overlap with DMSO peak), 46.2, 65.8, 75.6, 112.6, 114.9, 118.7, 127.8, 128.5, 129.5, 130.5, 136.5, 143.4, 150.4, 172.7; MS (ESI positive) *m/z* = 519.2 [M + H]^+^.

#### 2.2.29. (1-((4-Aminophenyl)sulfonyl)piperidin-4-yl)(4-benzhydrylpiperazin-1-yl)methanone (5g)

Compound **5g** was obtained in 70% yield; m.p. 174–176 °C; TLC: *R_f_* = 0.40 (ethyl acetate/*n-*hexane 70% v/v); ^1^H NMR (DMSO-d_6_, 400 MHz): *δ* 1.54 (2H, m, piperidine-C*H*_2_), 1.68 (2H, m, piperidine-C*H*_2_), 2.29 (6H, m, 2 × piperazine-C*H*_2,_ piperidine-C*H*_2_), 2.54 (1H, overlap with DMSO peak, COC*H*), 3.51 (6H, m, 2 × piperazine-C*H*_2_, piperidine-C*H*_2_), 4.32 (1H, s, C*H*), 6.06 (2H, s, exchange with D_2_O, N*H*_2_) 6.65 (2H, m, Ar-*H*), 6.91 (1H, m, Ar-*H*), 7.23 (3H, m, Ar-*H*), 7.30 (4H, t, *J* = 7.6, Ar-*H*), 7.44 (4H, d, *J* = 7.2, Ar-*H*); ^13^C NMR (DMSO-d_6_, 100 MHz): *δ* 28.6, 36.8, 40.4 (overlap with DMSO peak), 46.2, 62.2, 75.6, 113.6, 127.8, 128.5, 129.5, 130.5, 132.5, 143.4, 153.9, 172.7; MS (ESI positive) *m/z* = 519.2 [M + H]^+^.

#### 2.2.30. 2-((4-(bis(4-Fluorophenyl)methyl)piperazin-1-yl)sulfonyl)aniline (5h)

Compound **5h** was obtained in 94% yield; m.p. 176–179 °C (dec); TLC: *R_f_* = 0.46 (ethyl acetate/*n-*hexane 30% v/v); ^1^H NMR (DMSO-d_6_, 400 MHz): *δ* 2.41 (4H, m, 2 × piperazine-C*H*_2_), 3.03 (4H, m, 2 × piperazine-C*H*_2_), 4.44 (1H, s, C*H*), 6.07 (2H, s, exchange with D_2_O, N*H*_2_), 6.69 (1H, t, *J* = 8.0, Ar-*H*), 6.91 (1H, d, *J* = 8.0, Ar-*H*), 7.12 (4H, m, Ar-*H*), 7.39 (6H, m, Ar-H); ^13^C NMR (DMSO-d_6_, 100 MHz): *δ* 47.0, 51.1, 73.2, 115.4, 116.2 (d, ^2^*J*_C–F_ 21), 116.4, 118.2, 130.2 (d, ^3^*J*_C–F_ 8), 130.8, 135.2, 139.3, 148.4, 161.9 (d, ^1^*J*_C–F_ 242); ^19^F NMR (DMSO-d_6_, 376 MHz): *δ* –115.6 (2F, s); MS (ESI positive) *m/z* = 444.2 [M + H]^+^.

#### 2.2.31. 3-((4-(Bis(4-Fluorophenyl)methyl)piperazin-1-yl)sulfonyl)aniline (5i)

Compound **5i** was obtained in 53% yield; m.p. 183–186 °C (dec); TLC: *R_f_* = 0.21 (ethyl acetate/*n-*hexane 30% v/v); ^1^H NMR (DMSO-d_6_, 400 MHz): *δ* 2.39 (4H, m, 2 × piperazine-C*H*_2_), 2.91 (4H, m, 2 × piperazine-C*H*_2_), 4.42 (1H, s, C*H*), 5.69 (2H, s, exchange with D_2_O, N*H*_2_), 6.81 (1H, d, *J* = 7.8, Ar-*H*), 6.90 (2H, m, Ar-*H*), 7.12 (4H, m, Ar-*H*), 7.30 (1H, t, *J* = 7.8, Ar-*H*), 7.42 (4H, m, Ar-*H*); ^13^C NMR (DMSO-d_6_, 100 MHz): *δ* 47.0, 51.2, 73.2, 112.7, 115.1, 116.2 (d, ^2^*J*_C–F_ 21), 118.8, 130.2 (d, ^3^*J*_C–F_ 8), 130.5, 135.7, 139.2, 148.4, 161.9 (d, ^1^*J*_C–F_ 242); ^19^F NMR (DMSO-d_6_, 376 MHz): *δ* –115.6 (2F, s); MS (ESI positive) *m/z* = 444.2 [M + H]^+^.

#### 2.2.32. 4-((4-(Bis(4-Fluorophenyl)methyl)piperazin-1-yl)sulfonyl)aniline (5j)

Compound **5j** was obtained in 64% yield; m.p. 155–157 °C (dec); TLC: *R_f_* = 0.18 (ethyl acetate/*n-*hexane 30% v/v); ^1^H NMR (DMSO-d_6_, 400 MHz): *δ* 2.37 (4H, m, 2 × piperazine-C*H*_2_), 2.85 (4H, m, 2 × piperazine-C*H*_2_), 4.46 (1H, s, C*H*), 6.16 (2H, s, exchange with D_2_O, N*H*_2_), 6.71 (2H, d, *J* = 8.8, Ar-*H*), 7.12 (4H, m, Ar-*H*), 7.38 (2H, d, *J* = 8.8, Ar-*H*), 7.42 (4H, m, Ar-*H*); ^13^C NMR (DMSO-d_6_, 100 MHz): *δ* 47.0, 51.1, 73.2, 113.6, 116.2 (d, ^2^*J*_C–F_ 21), 125.6, 130.2 (d, ^3^*J*_C–F_ 8), 130.5, 139.2, 154.1, 161.9 (d, ^1^*J*_C–F_ 242); ^19^F NMR (DMSO-d_6_, 376 MHz): *δ* –115.6 (2F, s); MS (ESI positive) *m/z* = 444.2 [M + H]^+^.

#### 2.2.33. 2-Amino-N-(2-(4-(bis(4-fluorophenyl)methyl)piperazin-1-yl)-2-oxoethyl)benzenesulfonamide (5k)

Compound **5k** was obtained in 79% yield; m.p. 137–139 °C; TLC: *R_f_* = 0.16 (ethyl acetate/*n-*hexane 60% v/v); ^1^H NMR (DMSO-d_6_, 400 MHz): *δ* 2.24 (4H, m, 2 × piperazine-C*H*_2_), 3.33 (4H, m, overlap with water peak, 2 × piperazine-C*H*_2_), 3.66 (2H, s, COC*H_2_*NH) 4.42 (1H, s, C*H*), 5.99 (2H, s, exchange with D_2_O, N*H*_2_), 6.59 (1H, t, *J* = 7.2, Ar-*H*), 6.79 (2H, d, *J* = 8.0, Ar-*H*), 7.17 (4H, m, Ar-*H*), 7.28 (1H, m, Ar-*H*), 7.36 (1H, t, *J* = 7.2, exchange with D_2_O, SO_2_N*H*CH_2_), 7.46 (4H, m, Ar-*H*); ^13^C NMR (DMSO-d_6_, 100 MHz): *δ* 42.4, 45.1, 52.2, 75.5, 116.1, 116.3 (d, ^2^*J*_C–F_ 21), 116.6, 118.2, 130.2, (d, ^3^*J*_C–F_ 9), 130.8, 135.6, 139.2, 148.1, 162.1 (d, ^1^*J*_C–F_ 242), 171.3; ^19^F NMR (DMSO-d_6_, 376 MHz): *δ* –115.6 (2F, s); MS (ESI positive) *m/z* = 501.2 [M + H]^+^.

#### 2.2.34. 3-Amino-N-(2-(4-(bis(4-fluorophenyl)methyl)piperazin-1-yl)-2-oxoethyl)benzenesulfonamide (5l)

Compound **5l** was obtained in 32% yield; m.p. 162–164 °C; TLC: *R_f_* = 0.40 (ethyl acetate/*n-*hexane 70% v/v); ^1^H NMR (DMSO-d_6_, 400 MHz): *δ* 2.24 (4H, m, 2 × piperazine-C*H*_2_), 3.41 (4H, overlap with water peak, 2 × piperazine-C*H*_2_), 3.66 (2H, s, COC*H_2_*NH) 4.42 (1H, s, C*H*), 6.0 (2H, s, exchange with D_2_O, N*H*_2_), 6.77 (1H, d, *J* = 7.4, Ar-*H*), 6.90 (1H, d, *J* = 7.4, Ar-*H*), 6.99 (1H, s, Ar-*H*), 7.16 (5H, m, Ar-*H*), 7.46 (5H, m, 4 × Ar-*H*, exchange with D_2_O, SO_2_N*H*CH_2_); ^13^C NMR (DMSO-d_6_, 100 MHz): *δ* 42.4, 44.9, 51.9, 73.5, 112.0, 114.2, 116.9 (d, ^2^*J*_C–F_ 21), 118.1, 130.3 (d, ^3^*J*_C–F_ 8), 130.4, 139.2, 141.4, 150.1, 161.9 (d, ^1^*J*_C–F_ 242), 166.3; ^19^F NMR (DMSO-d_6_, 376 MHz): *δ* –115.6 (2F, s); MS (ESI positive) *m/z* = 501.2 [M + H]^+^.

#### 2.2.35. 4-Amino-N-(2-(4-(bis(4-fluorophenyl)methyl)piperazin-1-yl)-2-oxoethyl)benzenesulfonamide (5m)

Compound **5m** was obtained in 44% yield; m.p. 182–184 °C; TLC: *R_f_* = 0.28 (methanol/dichloromethane 5% v/v); ^1^H NMR (DMSO-d_6_, 400 MHz): *δ* 2.21 (4H, m, 2 × piperazine-C*H*_2_), 3.37 (4H, m, overlap with water peak, 2 × piperazine-C*H*_2_), 3.59 (2H, s, COC*H_2_*NH) 4.40 (1H, s, C*H*), 6.0 (2H, s, exchange with D_2_O, N*H*_2_), 6.61 (2H, d, *J* = 8.8, Ar-*H*), 7.16 (4H, m, Ar-*H*), 7.28 (1H, m, exchange with D_2_O, SO_2_N*H*CH_2_), 7.45 (6H, m, Ar-*H*); ^13^C NMR (DMSO-d_6_, 100 MHz): *δ* 42.4, 44.9, 51.7, 73.5, 113.4, 116.3 (*^2^J*_C–F_ 21), 125.6, 129.6, 130.3 (d, ^3^*J*_C–F_ 8), 139.2, 153.5, 162.0 (d, ^1^*J*_C–F_ 242), 166.6; ^19^F NMR (DMSO-d_6_, 376 MHz): *δ* –115.6 (2F, s); MS (ESI positive) *m/z* = 501.2 [M + H]^+^.

#### 2.2.36. (1-((2-Aminophenyl)sulfonyl)piperidin-4-yl)(4-(bis(4-fluorophenyl)methyl)piperazin-1-yl)methanone (5n)

Compound **5n** was obtained in 70% yield; m.p. 140–142 °C; TLC: *R_f_* = 0.35 (methanol/dichloromethane 5% v/v); ^1^H NMR (DMSO-d_6_, 400 MHz): *δ* 1.54 (2H, m, piperidine-C*H*_2_), 1.66 (2H, m, piperidine-C*H*_2_), 2.47 (4H, m, 2 × piperazine-C*H*_2_), 2.54 (3H, m, overlap with DMSO peak, piperidine-C*H*2, COC*H*), 3.46 (4H, m, 2 × piperazine-C*H*_2_), 3.64 (2H, m, piperidine-C*H*2), 3.42 (1H, s, C*H*), 6.06 (2H s, exchange with D_2_O, N*H*_2_), 6.66 (1H, t, *J* = 8.0, Ar-*H*), 6.87 (1H, d, *J* = 8.4, Ar-*H*), 7.16 (4H, t, *J* = 8.8, Ar-H), 7.38 (1H, m, Ar-*H*), 7.55 (4H, m, Ar-*H*), 7.59 (1H, m, Ar-*H*); ^13^C NMR (DMSO-d_6_, 100 MHz): *δ* 28.6, 36.8, 42.0, 45.9, 53.0, 75.6, 116.1, 116.3 (d, ^2^*J*_C–F_ 21), 116.6, 118.2, 130.2, (d, ^3^*J*_C–F_ 9), 130.8, 135.6, 139.2, 148.1, 162.1 (d, ^1^*J*_C–F_ 242), 171.3; ^19^F NMR (DMSO-d_6_, 376 MHz): *δ* –115.6 (2F, s); MS (ESI positive) *m/z* = 555.2 [M + H]^+^.

#### 2.2.37. (1-((3-Aminophenyl)sulfonyl)piperidin-4-yl)(4-(bis(4-fluorophenyl)methyl)piperazin-1-yl)methanone (5o)

Compound **5o** was obtained in 52% yield; m.p. 142–144 °C; TLC: *R_f_* = 0.31 (ethyl acetate/*n-*hexane 70% v/v); ^1^H NMR (DMSO-d_6_, 400 MHz): *δ* 1.54 (2H, m, piperidine-C*H*_2_), 1.68 (2H, m, piperidine-C*H*_2_), 2.23 (4H, m, 2 × piperazine-C*H*_2_), 2.33 (2H, m, piperidine-C*H*_2_), 2.60 (1H, m, COC*H*), 3.52 (4H, m, 2 × piperazine-C*H*_2_), 3.60 (2H, m, piperidine-C*H*_2_), 4.42 (1H, s, C*H*), 5.64 (2H, s, exchange with D_2_O, N*H*_2_), 6.82 (2H, m, Ar-*H*), 6.92 (1H, s, Ar-*H*), 7.16 (4H, m, Ar-*H*), 7.25 (1H, m, Ar-*H)*; 7.46 (4H, m, Ar-*H*); ^13^C NMR (DMSO-d_6_, 100 MHz): *δ* 28.6, 36.8, 41.9, 46.2, 52.4, 73.5, 112.6, 114.9, 116.2 (d, ^2^*J*_C–F_ 21), 118.7, 130.3 (d, ^3^*J*_C–F_ 8), 130.4, 136.5,139.2, 150.4, 162.0 (d, ^1^*J*_C–F_ 242), 172.6; ^19^F NMR (DMSO-d_6_, 376 MHz): *δ* –115.6 (2F, s); MS (ESI positive) *m/z* = 555.2 [M + H]^+^.

#### 2.2.38. (1-((4-Aminophenyl)sulfonyl)piperidin-4-yl)(4-(bis(4-fluorophenyl)methyl)piperazin-1-yl)methanone (5p)

Compound **5p** was obtained in 74% yield; m.p. 162–164 °C (dec); TLC: *R_f_* = 0.43 (ethyl acetate/*n-*hexane 70% v/v); ^1^H NMR (DMSO-d_6_, 400 MHz): *δ* 1.55 (2H, m, piperidine-C*H*_2_), 1.66 (2H, m, piperidine-C*H*_2_), 2.22 (6H, m, 2 × piperazine-C*H*_2,_ piperidine-C*H*_2_), 2.54 (1H, m, overlap with DMSO peak, COC*H*), 3.51 (6H, m, 2 × piperazine-C*H*_2,_ piperidine-C*H*_2_), 4.41 (1H, s, C*H*), 5.64 (2H, s, exchange with D_2_O, N*H*_2_), 6.66 (2H, d, *J* = 8.8, Ar-*H*), 7.16 (4H, m, Ar-*H*), 7.35 (2H, d, *J* = 8.8, Ar-*H*), 7.46 (4H, m, Ar-*H*); ^13^C NMR (DMSO-d_6_, 100 MHz): *δ* 28.6, 36.8, 41.9, 46.2, 52.4, 73.5, 113.5, 116.2 (d, ^2^*J*_C–F_ 21), 120.6, 130.2, 130.3 (d, ^3^*J*_C–F_ 8), 139.2, 153.9, 162.4 (d, ^1^*J*_C–F_ 242), 172.7; ^19^F NMR (DMSO-d_6_, 376 MHz): *δ* –115.6 (2F, s); MS (ESI positive) *m/z* = 555.2 [M + H]^+^.

### 2.3. General procedure for the synthesis of compounds 6a–p

The appropriate aminobenzensulfonamides **5a**–**p** (1.0 eq) dissolved in dry DMA (5.0 ml) at 0 °C were treated with Et_3_N (1.3 eq) and freshly prepared sulfamoyl chloride until consumption of starting material was confirmed (TLC monitoring). Then the solution was quenched with slush and extracted with EtOAc (3 × 20 ml). The combined organic layers were washed with NaHCO_3_ aqueous solution (3 × 10 ml), HCl aqueous solution 1.0 M (1 × 10 ml), brine (3 × 10 ml), dried over Na_2_SO_4_, filtered-off and concentrated under *vacuo*. The obtained residue was purified by trituration from Et_2_O to afford the titled sulfamides **6a**–**p** as white solids.

#### 2.3.1. 1-Benzhydryl-4-((3-sulfamoylaminophenyl)sulfonyl)piperazine (6a)

Compound **6a** was obtained in 14% yield; m.p. 125–127 °C; TLC: *R_f_* = 0.55 (ethyl acetate/*n-*hexane 60% v/v); ^1^H NMR (DMSO-d_6_, 400 MHz): *δ* 2.40 (4H, m, 2 × piperazine-C*H*_2_), 2.94 (4H, m, 2 × piperazine-C*H*_2_), 4.34 (1H, s, C*H*), 7.20 (2H, t, *J* = 7.2, Ar-*H*), 7.29 (5H, m, Ar-*H*), 7.40 (5H, m, Ar-*H*), 7.53 (3H, m, Ar-H, exchange with D_2_O, NHSO_2_N*H*_2_), 7.61 (1H, t, *J* = 7.6, Ar-*H*), 10.04 (1H, s, exchange with D_2_O, N*H*SO_2_NH_2_); ^13^C NMR (DMSO-d_6_, 100 MHz): *δ* 47.0, 51.4, 75.3, 117.3, 121.6, 122.7, 127.9, 128.4, 129.5, 129.6, 136.2, 141.3, 143.3; MS (ESI positive) *m/z* = 487.0 [M + H]^+^.

#### 2.3.2. 1-Benzhydryl-4-((4-sulfamoylaminophenyl)sulfonyl)piperazine (6b)

Compound **6b** was obtained in 42% yield; m.p. 207–210 °C; TLC: *R_f_* = 0.30 (ethyl acetate/*n-*hexane 50% v/v); ^1^H NMR (DMSO-d_6_, 400 MHz): *δ* 2.40 (4H, m, 2 × piperazine-C*H*_2_), 2.91 (4H, m, 2 × piperazine-C*H*_2_), 4.33 (1H, s, C*H*), 7.19 (2H, t, *J* = 7.2, Ar-*H*), 7.28 (4H, t, *J* = 7.2, Ar-*H*), 7.38 (8H, m, 6 × Ar-*H*, exchange with D_2_O, NHSO_2_N*H*_2_), 7.65 (2H, d, *J* = 8.4, Ar-*H*), 10.35 (1H, s, exchange with D_2_O, N*H*SO_2_NH_2_); ^13^C NMR (DMSO-d_6_, 100 MHz): *δ* 47.0, 51.3, 75.3, 117.5, 127.3, 127.9, 128.4, 129.5, 130.0, 143.3, 144.9; MS (ESI positive) *m/z* = 487.0 [M + H]^+^.

#### 2.3.3. N-(2-(4-Benzhydrylpiperazin-1-yl)-2-oxoethyl)-2-(sulfamoylamino)benzenesulfonamide (6c)

Compound **6c** was obtained in 10% yield; m.p. 150–152 °C; TLC: *R_f_* = 0.64 (ethyl acetate/*n-*hexane 60% v/v); ^1^H NMR (DMSO-d_6_, 400 MHz): *δ* 2.26 (4H, m, 2 × piperazine-C*H*_2_), 3.40 (4H, m, 2 × piperazine-C*H*_2_), 3.80 (2H, s, COC*H_2_*NH), 4.35 (1H, s, C*H*), 7.21 (3H, m, Ar-*H*), 7.33 (4H, m, Ar-*H*), 7.45 (4H, m, Ar-*H*), 7.50 (2H, s, exchange with D_2_O, NHSO_2_N*H*_2_), 7.64 (2H, m, Ar-*H*), 7.81 (1H, d, *J* = 8.0, Ar-*H*), 8.22 (1H, s, exchange with D_2_O, SO_2_N*H*CH_2_)_,_ 8.84 (1H, s, exchange with D_2_O, N*H*SO_2_NH_2_); ^13^C NMR (DMSO-d_6_, 100 MHz): *δ* 42.2, 45.3, 52.0, 75.6, 117.8, 123.1, 127.2, 127.9, 128.5, 129.5, 130.2, 134.6, 137.4, 143.3, 166.2; MS (ESI positive) *m/z* = 487.0 [M + H]^+^.

#### 2.3.4. N-(2-(4-Benzhydrylpiperazin-1-yl)-2-oxoethyl)-3-(sulfamoylamino)benzenesulfonamide (6d)

Compound **6d** was obtained in 29% yield; m.p. 154–156 °C; TLC: *R_f_* = 0.18 (methanol/dichloromethane 10% v/v); ^1^H NMR (DMSO-d_6_, 400 MHz): *δ* 2.26 (4H, m, 2 × piperazine-C*H*_2_), 3.13 (4H, m, 2 × piperazine-C*H*_2_), 3.72 (2H, s, COC*H_2_*NH), 4.34 (1H, s, C*H*), 7.24 (3H, m, Ar-*H*, exchange with D_2_O, SO_2_N*H*CH_2_), 7.33 (6H, m, Ar-*H*), 7.45 (7H, m, 5 × Ar-*H*, exchange with D_2_O, NHSO_2_N*H*_2_), 7.62 (1H, m, Ar-*H*), 8.99 (1H, s, exchange with D_2_O, N*H*SO_2_NH_2_); ^13^C NMR (DMSO-d_6_, 100 MHz): *δ* 42.2, 44.9, 51.9, 75.7, 116.4, 120.7, 122.5, 127.9, 128.6, 129.5, 130.5, 141.1, 141.6, 143.3, 171.3; MS (ESI positive) *m/z* = 544.0 [M + H]^+^.

#### 2.3.5. (4-Benzhydrylpiperazin-1-yl)(1-((2-sulfamoylaminophenyl)sulfonyl)piperidin-4-yl)methanone (6e)

Compound **6e** was obtained in 65% yield; m.p. 162–164 °C; TLC: *R_f_* = 0.41 (methanol/dichloromethane 10% v/v); ^1^H NMR (DMSO-d_6_, 400 MHz): *δ* 1.58 (2H, m, piperidine-C*H*_2_), 1.70 (2H, m, piperidine-C*H*_2_), 2.26 (4H, m, 2 × piperazine-C*H*_2_), 2.54 (2H, overlap with DMSO peak, piperidine-C*H*_2_), 2.60 (1H, overlap with DMSO peak, COC*H*), 3.47 (4H, m, 2 × piperazine-C*H*_2_), 3.65 (2H, m, piperidine-C*H*_2_), 4.33 (1H, s, C*H*), 7.23 (3H, m, Ar-*H*), 7.32 (5H, m, Ar-*H*), 7.43 (5H, m, Ar-*H*), 7.70 (3H, m, Ar-*H*, exchange with D_2_O, NHSO_2_N*H*_2_), 8.95 (1H, s, exchange with D_2_O, N*H*SO_2_NH_2_); ^13^C NMR (DMSO-d_6_, 100 MHz): *δ* 28.5, 36.6, 41.9, 46.0, 53.0, 75.6, 118.8, 121.7, 127.9, 128.6, 129.5, 130.9, 135.5, 138.3, 141.3, 143.3, 172.5; MS (ESI positive) *m/z* = 598.0 [M + H]^+^.

#### 2.3.6. (4-Benzhydrylpiperazin-1-yl)(1-((3-sulfamoylaminophenyl)sulfonyl)piperidin-4-yl)methanone (6f)

Compound **6f** was obtained in 65% yield; m.p. 230–232 °C; TLC: *R_f_* = 0.13 (methanol/dichloromethane 5% v/v); ^1^H NMR (DMSO-d_6_, 400 MHz): *δ* 1.58 (2H, m, piperidine-C*H*_2_), 1.68 (2H, m, piperidine-C*H*_2_), 2.25 (4H, m, 2 × piperazine-C*H*_2_), 2.33 (2H, m, piperidine-C*H*_2_), 2.54 (1H, overlap with DMSO peak, COC*H*), 3.4 (4H, m, 2 × piperazine-C*H*_2_), 3.59 (2H, m, piperidine-C*H*_2_), 4.32 (1H, s, C*H*), 7.21 (2H, m, Ar-*H*), 7.32 (7H, m, 5 × Ar-*H*, exchange with D_2_O, NHSO_2_N*H*_2_), 7.45 (5H, m, Ar-*H*), 7.54 (2H, m, Ar-*H*), 9.97 (1H, s, exchange with D_2_O, N*H*SO_2_NH_2_); ^13^C NMR (DMSO-d_6_, 100 MHz): *δ* 28.6, 36.8, 40.4 (overlap with DMSO peak), 46.2, 52.4, 75.6, 117.1, 121.3, 122.5, 126.2, 127.9, 128.5, 129.5, 130.8, 136.9, 141.3, 172.7; MS (ESI positive) *m/z* = 598.0 [M + H]^+^.

#### 2.3.7. (4-Benzhydrylpiperazin-1-yl)(1-((4-sulfamoylaminophenyl)sulfonyl)piperidin-4-yl)methanone (6g)

Compound **6 g** was obtained in 44% yield; m.p. 160–162 °C; TLC: *R_f_* = 0.26 (ethyl acetate/*n-*hexane 70% v/v); ^1^H NMR (DMSO-d_6_, 400 MHz): *δ* 1.55 (2H, m, piperidine-C*H*_2_), 1.67 (2H, m, piperidine-C*H*_2_), 2.25 (6H, m, 2 × piperazine-C*H*_2,_ piperidine-C*H*_2_), 2.54 (1H, m, overlap with DMSO peak, COC*H*), 3.47 (4H, m, 2 × piperazine-C*H*_2_), 3.58 (2H, m, piperidine-C*H*_2_), 4.31 (1H, s, C*H*), 7.21 (2H, t, *J* = 8.6, Ar-*H*), 7.32 (7H, m, Ar-*H*), 7.44 (4H, m, 2 × Ar-*H*, exchange with D_2_O, NHSO_2_N*H*_2_), 7.64 (2H, d, *J* = 8.2, Ar-*H*), 10.28 (1H, s, exchange with D_2_O, N*H*SO_2_NH_2_); ^13^C NMR (DMSO-d_6_, 100 MHz): *δ* 28.6, 36.9, 40.4 (overlap with DMSO peak), 46.2, 62.2, 75.6, 113.6, 127.8, 128.4, 129.5, 130.2, 132.6, 143.1, 153.9, 172.70; MS (ESI positive) *m/z* = 598.0 [M + H]^+^.

#### 2.3.8. 1-(Bis(4-Fluorophenyl)methyl)-4-((2-sulfamoylaminophenyl)sulfonyl)piperazine (6h)

Compound **6h** was obtained in 34% yield; m.p. 170–172 °C (dec); TLC: *R_f_* = 0.56 (methanol/dichloromethane 10% v/v); ^1^H NMR (DMSO-d_6_, 400 MHz): *δ* 2.41 (4H, m, 2 × piperazine-C*H*_2_), 3.03 (4H, m, 2 × piperazine-C*H*_2_), 4.44 (1H, s, C*H*), 7.13 (4H, m, Ar-*H*), 7.29 (1H, t, *J* = 7.4, Ar-*H*), 7.43 (5H, m, Ar-*H*) , 7.75 (4H, m, 2 × Ar-*H*, exchange with D_2_O, NHSO_2_N*H*_2_), 8.95 (1H, s, exchange with D_2_O, N*H*SO_2_NH_2_); ^13^C NMR (DMSO-d_6_, 100 MHz): *δ* 46.8, 51.1, 73.1, 115.9, 116.2 (d, ^2^*J*_C–F_ 21), 118.3, 123.0, 130.2 (d, ^3^*J*_C–F_ 7), 130.8, 135.1, 138.6, 139.1, 161.9 (d, ^1^*J*_C–F_ 241); ^19^F NMR (DMSO-d_6_, 376 MHz): *δ* –115.4 (2F, s); MS (ESI positive) *m/z* = 523.0 [M + H]^+^.

#### 2.3.9. 1-(Bis(4-Fluorophenyl)methyl)-4-((3-sulfamoylaminophenyl)sulfonyl)piperazine (6i)

Compound **6i** was obtained in 43% yield; m.p. 132–135 °C; TLC: *R_f_* = 0.30 (ethyl acetate/*n-*hexane 50% v/v); ^1^H NMR (DMSO-d_6_, 400 MHz): *δ* 2.39 (4H, m, 2 × piperazine-C*H*_2_), 2.96 (4H, m, 2 × piperazine-C*H*_2_), 4.43 (1H, s, C*H*), 7.12 (4H, t, *J* = 8.8, Ar-*H*), 7.37 (7H, m, 5 × Ar-*H,* exchange with D_2_O, NHSO_2_N*H*_2_), 7.52 (2H, m, Ar-*H*), 7.59 (1H, m, Ar-*H*), 10.01 (1H, s, exchange with D_2_O, N*H*SO_2_NH_2_); ^13^C NMR (DMSO-d_6_, 100 MHz): *δ* 47.0, 51.2, 73.2, 116.2 (d, ^2^*J*_C–F_ 21), 117.2, 121.5, 122.7, 130.2 (d, ^3^*J*_C–F_ 8), 130.8, 136.1, 139.2, 141.3, 161.9 (d, ^1^*J*_C–F_ 242); ^19^F NMR (DMSO-d_6_, 376 MHz): *δ* –115.6 (2F, s); MS (ESI positive) *m/z* = 523.0 [M + H]^+^.

#### 2.3.10. 1-(Bis(4-Fluorophenyl)methyl)-4-((4-sulfamoylaminophenyl)sulfonyl)piperazine (6j)

Compound **6j** was obtained in 42% yield; m.p. 176–179 °C (dec); TLC: *R_f_* = 0.28 (methanol/dichloromethane 10% v/v); ^1^H NMR (DMSO-d_6_, 400 MHz): *δ* 2.38 (4H, m, 2 × piperazine-C*H*_2_), 2.90 (4H, m, 2 × piperazine-C*H*_2_), 4.42 (1H, s, C*H*), 7.12 (4H, m, Ar-*H*), 7.41 (8H, m, 6 × Ar-*H*, exchange with D_2_O, NHSO_2_N*H*_2_), 7.65 (2H, d, *J* = 8.4, Ar-*H*), 10.33 (1H, s, exchange with D_2_O, N*H*SO_2_NH_2_); ^13^C NMR (DMSO-d_6_, 100 MHz): *δ* 47.0, 51.1, 73.2, 116.2 (d, ^2^*J*_C–F_ 21), 117.5, 127.2, 129.9, 130.2 (d, ^3^*J*_C–F_ 8), 139.2, 144.8, 161.9 (d, ^1^*J*_C–F_ 242); ^19^F NMR (DMSO-d_6_, 376 MHz): *δ* –115.6 (2F, s); MS (ESI positive) *m/z* = 523.0 [M + H]^+^.

#### 2.3.11. N-(2-(4-(bis(4-Fluorophenyl)methyl)piperazin-1-yl)-2-oxoethyl)-2-(sulfamoylamino)benzenesulfonamide (6k)

Compound **6k** was obtained in 80% yield; m.p. 183–186 °C (dec); TLC: *R_f_* = 0.23 (methanol/dichloromethane 5% v/v); ^1^H NMR (DMSO-d_6_, 400 MHz): *δ* 2.20 (4H, m, 2 × piperazine-C*H*_2_), 3.45 (4H, m, 2 × piperazine-C*H*_2_), 3.75 (2H, s, COC*H_2_*NH) 4.43 (1H, s, C*H*), 7.17 (5H, m, Ar-*H*), 7.46 (6H, m, 4 × Ar-*H*, exchange with D_2_O, NHSO_2_N*H*_2_), 7.63 (2H, m, Ar-*H*), 7.80 (1H, d, *J* = 8.0, Ar-*H*), 8.23 (1H, m, exchange with D_2_O, SO_2_N*H*CH_2_), 8.84 (1H, s, exchange with D_2_O, N*H*SO_2_NH_2_); ^13^C NMR (DMSO-d_6_, 100 MHz): *δ* 42.4, 44.9, 51.9, 73.5, 116.3 (d, ^2^*J*_C–F_ 21), 116.7, 118.2, 123.1, 130.3 (d, ^3^*J*_C–F_ 10), 130.5, 134.9, 138.2, 139.3, 162.0 (d, ^1^*J*_C–F_ 242), 171.4; ^19^F NMR (DMSO-d_6_, 376 MHz): *δ* –115.6 (2F, s); MS (ESI positive) *m/z* = 580.0 [M + H]^+^.

#### 2.3.12. N-(2-(4-(bis(4-Fluorophenyl)methyl)piperazin-1-yl)-2-oxoethyl)-3-(sulfamoylamino)benzenesulfonamide (6l)

Compound **6l** was obtained in 29% yield; m.p. 155–157 °C (dec); TLC: *R_f_* = 0.28 (methanol/dichloromethane 5% v/v); ^1^H NMR (DMSO-d_6_, 400 MHz): *δ* 2.27 (4H, m, 2 × piperazine-C*H*_2_), 3.41 (4H, overlap with water peak, 2 × piperazine-C*H*_2_), 3.76 (2H, s, COC*H_2_*NH) 4.43 (1H, s, C*H*), 7.16 (5H, m, Ar-*H*), 7.26 (2H, s, exchange with D_2_O, NHSO_2_N*H*_2_), 7.36 (1H, m, exchange with D_2_O, SO_2_N*H*CH_2_), 7.42 (6H, m, Ar-*H*), 7.61 (1H, s, Ar-*H*), 9.93 (1H, s, exchange with D_2_O, N*H*SO_2_NH_2_); ^13^C NMR (DMSO-d_6_, 100 MHz): *δ* 42.4, 44.9, 51.9, 73.5, 116.9 (d, ^2^*J*_C–F_ 21), 118.4, 122.3, 123.2, 130.4 (d, ^3^*J*_C–F_ 8), 130.9, 137.2, 139.1, 141.2, 161.9 (d, ^1^*J*_C–F_ 242), 167.2; ^19^F NMR (DMSO-d_6_, 376 MHz): *δ* –115.6 (2F, s); MS (ESI positive) *m/z* = 580.0 [M + H]^+^.

#### 2.3.13. N-(2-(4-(bis(4-Fluorophenyl)methyl)piperazin-1-yl)-2-oxoethyl)-4-(sulfamoylamino)benzene sulfonamide (6m)

Compound **6m** was obtained in 35% yield; m.p. 157–159 °C; TLC: *R_f_* = 0.12 (methanol/dichloromethane 5% v/v); ^1^H NMR (DMSO-d_6_, 400 MHz): *δ* 2.22 (4H, m, 2 × piperazine-C*H*_2_), 3.37 (4H, m, overlap with water peak, 2 × piperazine-C*H*_2_), 3.68 (2H, s, COC*H_2_*NH), 4.42 (1H, s, C*H*), 7.16 (4H, m, Ar-*H*), 7.22 (2H, d, *J* = 8.8, Ar-*H*), 7.40 (2H, s, exchange with D_2_O, NHSO_2_N*H*_2_), 7.47 (5H, m, 4 × Ar-*H*, exchange with D_2_O, SO_2_N*H*CH_2_), 7.76 (2H, d, *J* = 8.8, Ar-*H*), 10.18 (1H, s, exchange with D_2_O, N*H*SO_2_NH_2_); ^13^C NMR (DMSO-d_6_, 100 MHz): *δ* 42.4, 44.9, 51.7, 73.5, 116.3 (*^2^J*_C–F_ 21), 116.6, 129.6, 130.1, 130.3 (d, ^3^*J*_C–F_ 8), 139.3, 142.9, 162.0 (d, ^1^*J*_C–F_ 242), 166.6; ^19^F NMR (DMSO-d_6_, 376 MHz): *δ* –115.6 (2F, s); MS (ESI positive) *m/z* = 580.0 [M + H]^+^.

#### 2.3.14. (4-(bis(4-Fluorophenyl)methyl)piperazin-1-yl)(1-((2-sulfamo-ylaminophenyl)sulfonyl)piperidin-4-yl)methanone (6n)

Compound **6n** was obtained in 44% yield; m.p. 140–142 °C; TLC: *R_f_* = 0.50 (methanol/dichloromethane 5% v/v); ^1^H NMR (DMSO-d_6_, 400 MHz): *δ* 1.56 (2H, m, piperidine-C*H*_2_), 1.70 (2H, m, piperidine-C*H*_2_), 2.24 (4H, m, 2 × piperazine-C*H*_2_), 2.58 (2H, m, piperidine-C*H*_2_), 2.68 (1H, m, COC*H*), 3.48 (4H, m, 2 × piperazine-C*H*_2_), 3.66 (2H, m, piperidine-C*H*_2_), 4.42 (1H, s, C*H*), 7.22 (5H, m, Ar-*H*), 7.43 (4H, m, Ar-*H*), 7.60 (2H, s, exchange with D_2_O, NHSO_2_N*H*_2_), 7.65 (2H, m, Ar-*H*), 7.76 (1H, m, Ar-*H*), 8.95 (1H, s, exchange with D_2_O, N*H*SO_2_NH_2_); ^13^C NMR (DMSO-d_6_, 100 MHz): *δ* 28.6, 36.8, 41.9, 46.2, 52.4, 73.5, 115.1, 115.4 (d, ^2^*J*_C–F_ 21), 118.0, 123.0, 129.4 (d, ^3^*J*_C–F_ 8), 130.8, 135.4, 138.4, 139.6, 161.1 (d, ^1^*J*_C–F_ 242), 168.4; ^19^F NMR (DMSO-d_6_, 376 MHz): *δ* –115.6 (2F, s); MS (ESI positive) *m/z* = 634.0 [M + H]^+^.

#### 2.3.15. (4-(bis(4-Fluorophenyl)methyl)piperazin-1-yl)(1-((3-sulfamoylaminophenyl)sulfonyl)piperidin-4-yl)methanone (6o)

Compound **6o** was obtained in 35% yield; m.p. 160–162 °C (dec); TLC: *R_f_* = 0.12 (methanol/dichloromethane 5% v/v); ^1^H NMR (DMSO-d_6_, 400 MHz): *δ* 1.54 (2H, m, piperidine-C*H*_2_), 1.68 (2H, m, piperidine-C*H*_2_), 2.23 (4H, m, 2 × piperazine-C*H*_2_), 2.33 (2H, m, piperidine-C*H*_2_), 2.54 (1H, m, overlap with DMSO peak, COC*H*), 3.47 (4H, m, 2 × piperazine-C*H*_2_), 3.60 (2H, m, piperidine-C*H*_2_), 4.42 (1H, s, C*H*), 7.16 (5H, m, Ar-*H*), 7.32 (2H, s, exchange with D_2_O, NHSO_2_N*H*_2_), 7.54 (7H, m, Ar-*H*), 9.81 (1H, s, exchange with D_2_O, N*H*SO_2_NH_2_); ^13^C NMR (DMSO-d_6_, 100 MHz): *δ* 28.6, 36.8, 41.9, 46.2, 52.4, 73.5, 116.2 (d, ^2^*J*_C–F_ 21), 118.7, 122.0, 122.9, 130.3 (d, ^3^*J*_C–F_ 8), 130.9, 135.3, 137.4, 139.6, 162.0 (d, ^1^*J*_C–F_ 242), 172.6; ^19^F NMR (DMSO-d_6_, 376 MHz): *δ* –115.6 (2F, s); MS (ESI positive) *m/z* = 634.0 [M + H]^+^.

#### 2.3.16. (4-(bis(4-Fluorophenyl)methyl)piperazin-1-yl)(1-((4-sulfamo-ylaminophenyl)sulfonyl)piperidin-4-yl)methanone (6p)

Compound **6p** was obtained in 46% yield; m.p. 150–152 °C (dec); TLC: *R_f_* = 0.16 (methanol/dichloromethane 5% v/v); ^1^H NMR (DMSO-d_6_, 400 MHz): *δ* 1.55 (2H, m, piperidine-C*H*_2_), 1.67 (2H, m, piperidine-C*H*_2_), 2.26 (6H, m, 2 × piperazine-C*H*_2,_ piperidine-C*H*_2_), 2.54 (1H, m, overlap with DMSO peak, COC*H*), 3.46 (4H, m, 2 × piperazine-C*H*_2_), 3.59 (2H, m, piperidine-C*H*_2_), 4.41 (1H, s, C*H*), 7.15 (4H, t, *J* = 8.6, Ar-*H*), 7.32 (2H, d, *J* = 8.2, Ar-*H*), 7.46 (6H, m, 4 × Ar-*H*, exchange with D_2_O, NHSO_2_N*H*_2_), 7.64 (2H, d, *J* = 8.2, Ar-*H*) 10.28 (1H, s, exchange with D_2_O, N*H*SO_2_NH_2_); ^13^C NMR (DMSO-d_6_, 100 MHz): *δ* 28.6, 36.8, 41.9, 46.2, 52.4, 73.5, 116.2 (d, ^2^*J*_C–F_ 21), 116.6, 129.7, 130.2, 130.3 (d, ^3^*J*_C–F_ 8), 139.2, 143.1, 162.4 (d, ^1^*J*_C–F_ 242), 172.70; ^19^F NMR (DMSO-d_6_, 376 MHz): *δ* –115.6 (2F, s); MS (ESI positive) *m/z* = 634.0 [M + H]^+^.

### 2.4. Carbonic anhydrase inhibition assay

An Applied Photophysics (Leatherhead, UK) stopped-flow instrument has been used for assaying the CA-catalysed CO_2_ hydration activity^18^. Phenol red (at a concentration of 0.2 mM) has been used as indicator, working at the absorbance maximum of 557 nm, with 20 mM Hepes (pH 7.5) as buffer, and 20 mM Na_2_SO_4_ (for maintaining constant the ionic strength), following the initial rates of the CA-catalysed CO_2_ hydration reaction for a period of 10–100 s. The CO_2_ concentrations ranged from 1.7 to 17 mM for the determination of the kinetic parameters and inhibition constants. For each inhibitor, at least six traces of the initial 5–10% of the reaction have been used for determining the initial velocity. The uncatalysed rates were determined in the same manner and subtracted from the total observed rates. Stock solutions of inhibitor (0.1 mM) were prepared in distilled-deionised water and dilutions up to 0.01 nM were done thereafter with the assay buffer. Inhibitor and enzyme solutions were preincubated together for 15 min at room temperature prior to assay, in order to allow for the formation of the E–I complex. The inhibition constants were obtained by non-linear least-squares methods using PRISM 3 and the Cheng-Prusoff equation, as reported earlier, and represent the mean from at least three different determinations^19^^,^^20^. All CA isoforms were recombinant ones obtained in-house as reported earlier^21^.

## 3. Results and discussion

### 3.1. Chemistry

The sulfamide moiety is able to establish a more extended network of hydrogen bonds within the enzymatic pocket compared to classical sulfonamides, due to the presence of the additional nitrogen atom, but unfortunately this feature does not confer selectivity against different CA isoforms^16^^,^^17^. Therefore, the purpose of this study is not only to explore the activity of the sulfamides against the two CA isoforms of *Vibrio cholerae*, but also to propose specific structural changes to obviate to the problem of selectivity.

**Scheme 1.**
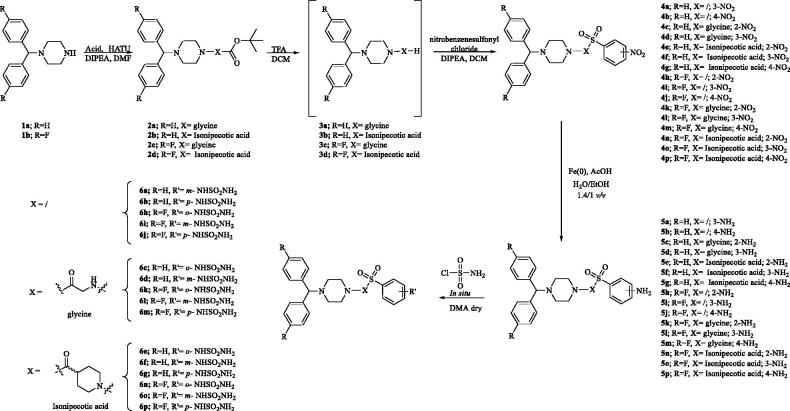
General synthetic scheme of compounds **6a**–**p**.

The novel sulfamides reported here feature the sulfamides groups (in *ortho*-, *meta*-, or *para* positions on the benzene ring), connected into a highly flexible alkyl-aryl scaffolds. In particular, we designed two series of compounds which differ by the spacer connecting the 1-benzhydrylpiperazin tail with the benzene-sulfamide zinc binding functionality (Scheme 1)^16^.

**Figure 1.**
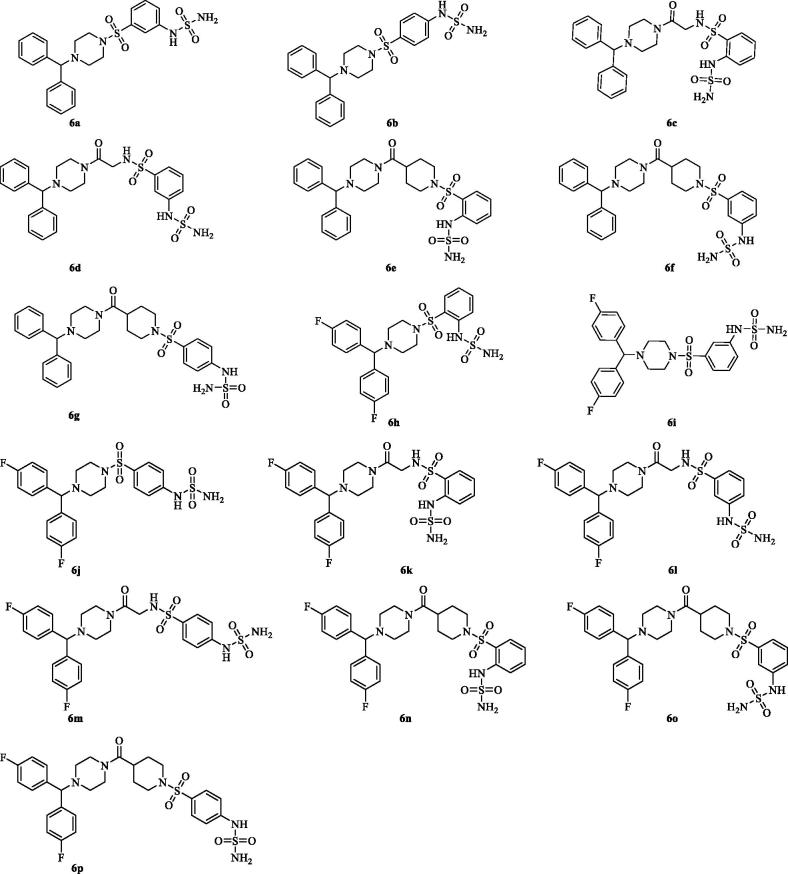
Structures of the compounds **6a**–**p**.

The planned synthesis of the compounds **6a**–**p** provides a first step during which the coupling of the benzhydryl piperazines **1a**,**b** with the appropriate acids takes place to give the intermediates **2a**–**d**, followed by treatment with trifluoroacetic acid (TFA) to afford the alkylamines **3a**–**d**. In the second step, the free amines were coupled with commercially available nitrobenzenesulfonyl chlorides and the obtained nitro derivatives **4a**–**p** were reduced with iron, Fe(0), in acidic media, to afford the amines **5a**–**p**. Finally, the desired compounds **6a**–**p** (Figure 1) were obtained by treatment of amines **5a**–**p** with freshly prepared sulfamoyl chloride^16^. All compounds were purified by trituration from Et_2_O to afford products with high purity in yields between 10 and 80%; the analytical and spectroscopic data of the purified compounds are in agreement with the purposed structures (see section 2 for details).

### 3.2. Carbonic anhydrase inhibition

All synthesised compounds **6a**–**p**, were tested *in vitro* for their inhibitory activity against the cytosolic isoenzymes hCA I and II and bacterial enzymes VchCAα and VchCAβ by means of a stopped-flow carbon dioxide hydration assay^18^, and their activities were compared to the standard CAI acetazolamide (AAZ). The following structure–activity–relationship (SARs) can be drawn from the data of Table 1:

**Table 1.** Inhibition data of hCA I, hCA II, VchCA α, VchCA β with compounds reported here and the standard sulfonamide inhibitor acetazolamide (**AAZ**) by a stopped flow CO_2_ hydrase assay^18^.

*K*_I_ (nM)*				
Cmp	hCA I	hCA II	VchCAα	VchCAβ
**6a**	66.2	417.4	333.7	4676
**6b**	555.1	359.2	236.4	1881
**6c**	839.9	850.2	930.8	>10,000
**6d**	58.1	396.4	95.7	3783
**6e**	578.1	1360	879.3	>10,000
**6f**	43.1	576.4	654.2	3792
**6g**	601.3	226.7	140.3	2538
**6h**	842.6	1171	2797.0	>10,000
**6i**	75.0	389.2	588.0	1742
**6j**	64.6	352.9	273.8	4349
**6k**	337.8	1431	1747.0	>10,000
**6l**	71.1	217.5	91.4	>10,000
**6m**	47.8	207.8	105.6	4666
**6n**	682.6	589.8	3748.6	>10,000
**6o**	72.0	663.1	497.2	1578
**6p**	49.1	181.7	398.2	4534
**AAZ**	250	12	6.8	451

*Mean from three different assays, by a stopped flow technique (errors were in the range of ±5–10% of the reported values).

- hCA I was effectively inhibited by most compounds investigated here, with inhibition constants spanning between 43.1 and 839.9 nM. It is interesting to note the case of compounds **6m** and **6p** that demonstrate the best activity (*K*_I_s = 47.8 and 49.1 nM), both having the sulfamide group in *para* position of the aromatic ring but differing for the glycine and isonipecotic acid spacer, respectively. The removal of the fluorine atoms in the aromatic rings of compound **6g** (corresponding to the non-halogenated analogue of **6d**), decreased the activity (*K*_I_ = 601.3 nM), suggesting that substitution with halogens as –F, in this case, is advantageous for the inhibitory activity. The same trend concerned compounds **6j** and its corresponding non-halogenated **6b**, which showed the following inhibition data *K*_I_s of 64.6 and 555.1 nM, respectively. On the other hand, the presence of fluorine atoms in aromatic rings of compound **6i** (*K*_I_ = 75.0 nM), did not have a significant impact on its activity, both compound having a similar activity to that of **6a** (*K*_I_ = 66.2 nM). The inhibition potencies were dependent not only on the presence of halogens but also on the sulfamide regioisomer considered. In fact, the potency ranking for compounds **6j**, **6i**, and **6h** (*K*_I_s = 64.6, 75.0, 842.6 nM), **6m**, **6l**, and **6k** (*K*_I_s = 47.8, 71.1, 337.8 nM) and for compounds **6p**, **6o**, and **6n** (*K*_I_s = 49.1, 72.0, 682.6 nM) was *para*>*meta*>*ortho*.
- The ubiquitous hCA II was inhibited by sulfamides **6a**–**p**, with *K*_I_s spanning between range 181.7 and 1430.7 nM. Likewise hCA I, the derivatives with sulfamide group in *para* position of the aromatic ring showed better activity than the corresponding *meta* and *ortho* analogues. In fact, compound **6g** shows a lower *K*_I_ = 226.7 nM than its *meta*-substituted analogue **6f** (*K*_I_ = 576.4 nM) or *ortho*-substituted derivative **6e** (*K*_I_ = 1360.1 nM). The introduction of fluorine atoms on the phenyl rings of compounds **6g**, **6f**, and **6e** to give derivatives **6o**, **6p**, and **6n**, caused a slight change in the inhibition trend, with a better *K*_I_ of the *ortho*-substituted compared to the *meta*-substituted derivative. It is interesting to underline the case of the compound **6p** (*K*_I_ = 181.7 nM), which is the best inhibitor against hCA II. The substitution of the isonipecotic acid spacer of **6p** with the glycine spacer of **6m** did not show any impact on the inhibitory activity. The removal of the fluorine atoms in **6g** did not substantially altered the activity compared to the perhydro derivative. Equally to hCA I, for hCA II the most considerable differences of inhibitory activity concerned the position of the ZBG in the phenyl ring, as demonstrated for example by compounds **6d** (*meta*-substituted) and **6c** (*ortho*-substituted) with *K*_I_s of 396.4 and 850.2 nM, respectively.
- The general tendencies described above were also applicable for the VchCAα, with this latter being moderately inhibited by benzenesulfamide derivatives prepared in this study (*K*_I_s values in the range of 91.4 and 3748.6 nM). As reported for the human isoforms discussed above, also for VchCAα, the worst inhibitors are reconfirmed to be the compounds that present the ZBG in the *ortho* position of the phenyl ring (**6c**, **6e**, **6h**, **6k**, and **6n** with *K*_I_s = 930.8, 879.3, 2797.0, 1747.0, and 3748.6 nM, respectively). The most active compounds were the halogen derivative **6l** and the non-halogen analogue **6d**, which exhibited *K*_I_ values of 91.4 and 95.7 nM, respectively. It is interesting to note that the introduction of the rigid linker of isonipecotic acid determined a lower efficacy against this isoform, as shown by the compounds **6f** (*K*_I_ = 654.2 nM) and **6o** (*K*_I_ = 497.2 nM), compared with the above-mentioned derivatives **6d** and **6l**. This improvement of VchCAα inhibition potency might be due to the flexible glycine spacer, which probably increases the interactions of the inhibitor with active site amino acids residues.
- VchCAβ was the least inhibited among the enzymes herein considered and showed *K*_I_s spanning between 1578.2 and 4676.1 nM. Interestingly, the *meta* derivatives **6o** and **6i** (*K*_I_s = 1578.2 and 1742.2 nM) were the most potent inhibitors against the VchCAβ, compared to the corresponding *para* analogues **6p** and **6j** (*K*_I_s = 4533.7 and 4349.2 nM). On the contrary, the *meta* derivatives **6a** and **6f** (*K*_I_s = 4676.1 and 3792.0 nM) were less effective with respect to *para* derivatives **6b** and **6g** (*K*_I_s = 1880.7 and 2538.1 nM). It is rather difficult to explain this result but we could speculate that the introduction of fluorine atoms leads to a decrease in the inhibitory potency of sulfamides against VchCAβ. In fact the fluorination of compounds **6g**, **6b**, and **6d** (*K*_I_s = 2538.1, 1880.7, and 3782.8 nM), to afford **6p**, **6j**, and **6l**, respectively, decreased the inhibition efficacy (*K*_I_s = 4533.7, 4349.2, and >10,000 nM, respectively), with the potency of compound **6l** being very low. Finally, it is important to emphasise the selectivity of some compounds against VchCAα with respect to the β-bacterial isoform. For instance, compounds **6l** and **6d** showed a nanomolar efficacy against VchCAα (*K*_I_s of 91.4 and 95.7 nM) but **6l** was totally ineffective against the β-isoform, whereas compound **6d** showed a *K*_I_ of 3782.8 nM against VchCAβ.

## 4. Conclusions

In this work, we reported the investigation of benzenesulfamide derivatives (–NH-SO_2_NH_2_) as an alternative zinc binding class of inhibitors to the primary aromatic sulfonamides in the design of new potential bacterial CAIs^17^. The “tail approach”, the method that consists in varying the terminal portions of the molecule to selectively enhance the inhibition of the target isoform(s), was applied to the phenyl sulfamide scaffold. All compounds were synthesised by a convenient and efficient method and were screened for *in vitro* inhibition against cytosolic isoenzymes hCA I, II, VchCAα and VchCAβ by using a stopped-flow CO_2_ hydrase assay method. The obtained results suggest that the benzhydryl piperazine moiety appended to benzene-sulfamide functionalities are not favourable for the interaction with *Vibrio cholerae* CA isoforms. Conversely, the same moiety is slightly more favourable for the inhibition of hCA I, as demonstrated by compounds **6f**, **6m**, **6p**, and **6d** that showed interesting activities against this isoform, with a nanomolar inhibition constant (*K*_I_s = 43.1, 47.8, 49.1, and 58.1 nM, respectively), being thus more potent than AAZ (as standard inhibitor).
